# Pulse pressure and diabetes treatments: Blood pressure and pulse pressure difference among glucose lowering modality groups in type 2 diabetes

**DOI:** 10.1097/MD.0000000000009791

**Published:** 2018-02-09

**Authors:** Hamid Alemi, Pegah Khaloo, Mohammad Ali Mansournia, Soghra Rabizadeh, Salome Sadat Salehi, Hossein Mirmiranpour, Neda Meftah, Alireza Esteghamati, Manouchehr Nakhjavani

**Affiliations:** a Endocrinology and Metabolism Research Center (EMRC), Vali-Asr Hospital, School of Medicine; b Department of Epidemiology and Biostatistics, School of Public Health, Tehran University of Medical Sciences, Tehran, Iran.

**Keywords:** cardiovascular safety, glibenclamide, insulin, metformin, pulse pressure, systolic and diastolic blood pressure, type 2 diabetes mellitus

## Abstract

Type 2 diabetes is associated with higher pulse pressure. In this study, we assessed and compared effects of classic diabetes treatments on pulse pressure (PP), systolic blood pressure (SBP), and diastolic blood pressure (DBP) in patients with type 2 diabetes.

In a retrospective cohort study, 718 non-hypertensive patients with type 2 diabetes were selected and divided into 4 groups including metformin, insulin, glibenclamide+metformin, and metformin+insulin. They were followed for 4 consecutive visits lasting about 45.5 months. Effects of drug regimens on pulse and blood pressure over time were assessed separately and compared in regression models with generalized estimating equation method and were adjusted for age, duration of diabetes, sex, smoking, and body mass index (BMI).

Studied groups had no significant change in PP, SBP, and DBP over time. No significant difference in PP and DBP among studied groups was observed (PP:P = 0.090; DBP:P = 0.063). Pairwise comparisons of PP, SBP, and DBP showed no statistically significant contrast between any 2 studied groups. Interactions of time and treatment were not different among groups.

Our results demonstrate patients using metformin got higher PP and SBP over time. Averagely, pulse and blood pressure among groups were not different. Trends of variation in pulse and blood pressure were not different among studied diabetes treatments.

## 1 Introduction

Pulse pressure (PP), defined as the difference between systolic blood pressure (SBP) and diastolic blood pressure (DBP) is the clinical manifestation of arterial stiffness.^[1]^ The systolic component of a wide PP increases the cardiac demand by exerting higher afterload on the heart which results in myocardial hypertrophy; while its diastolic element limits cardiac supply by decreasing coronary perfusion.^[2]^ Hence, wide PP is associated with the incidence of cardiovascular disease (CVD) besides the occurrence of cerebrovascular disease and nephropathy.^[3–10]^ PP is known to be higher in diseases involving vascular system such as the type 2 diabetes mellitus (DM2). People with DM2 have increased arterial stiffness resulting in a wide PP compared with their non-diabetic peers.^[7]^ The risk of CVD is higher in DM2 patients; furthermore, increase in their PP is an additional risk factor for CVD incidence and has a positive association with mortality.^[11]^

Drugs used to control the blood glucose in DM2 also could modify blood pressure as their side effects^[12–14]^; however, outcomes are not the same. For instance, while metformin could reduce blood pressure, insulin treatment does not have the similar effect.^[15–19]^ Also, even there is not a consensus over the general effect of some antidiabetic agents like metformin and insulin on blood pressure.^[19–23]^

Although several works have been done on the effect of different glucose-lowering therapies on blood pressure; little is known about their action on PP. Along with their study, Skov et al^[24]^ demonstrate that there is no statistically significant difference between PP of diabetic patients treated with insulin and its combinations with metformin (Met) or Rosiglitazone or both. We have postulated there should not be any difference between the effect of studied hypoglycemic treatments on pulse and blood pressure of diabetic patients. However, yet, there is not a comprehensive study comparing classic treatments applied in DM2. Moreover, there are some confounding factors need to be studied. Therefore, in the current study, we assessed and compared the effect of glucose-lowering regimens including metformin, Met+insulin, glibenclamide+metformin (Glb+Met), and insulin on diabetic patients PP, SBP, and DBP considering confounding factors over 45.5 months of follow-up.

## 2 Materials and methods

### 2.1 Study sample

This study is a part of an open prospective cohort conducted in the diabetes clinic of Valiasr hospital (Tehran, Iran). Data collection for this cohort started from 2008. Patients with DM2 who had attended Valiasr diabetes clinic have been enrolled in the original cohort. In the current study, we selected 718 diabetic non-hypertensive patients who had been already using metformin, Glb+Met, Met+insulin, or insulin only from the ongoing cohort study.

We excluded patients with primary hypertension as well as patients treated with lipid-lowering therapies other than statins. For avoiding the bias resulted by excluding normotensive people who got hypertensive later along follow-up, we traced those individuals who were totally 13 people (6 patients on metformin, 2 metformin and glibenclamide combination, 1 using the combination of insulin and metformin, and 1 patient using insulin). Patients with uncontrolled hypothyroidism or clinical hyperthyroidism were not included in the study as well as patients with any other interfering endocrinological disease. Those individuals with any history of myocardial infarction, percutaneous coronary intervention, stent placement, CCU admission, and cerebrovascular accidents were excluded from the study.

Patients were followed at the Valiasr hospital from the date of enrollment (the baseline visit) every year for 3 consecutive visits until October 2015 which had been set as the end of the follow-up. At each follow-up session, we included only patients who did not have any change in the drug which had been previously used.

Before enrollment, written informed consents were taken from all participants. The ethics committee of the Tehran University of Medical Sciences approved the study protocol.

### 2.2 Clinical and laboratory measurements

All the patient's characteristics, including age, duration of DM2 diagnosis, sex, body weight, height, body mass index (BMI), blood pressure, total cholesterol, high-density lipoprotein cholesterol, low-density lipoprotein (LDL) cholesterol, triglycerides, HbA1c, fasting blood glucose (FBS), 1hour post-prandial glucose (1hppg), creatinine concentration, and medication (antihypertensive drug, cholesterol-lowering drug, and antidiabetic drug) were extracted from the computerized hospitalization records at the baseline and during the follow-up.

Age, medication, and the duration of DM2 have been obtained from the participants through the interview at the first visit. Weight, height, and the waist circumference were measured at the baseline. Systolic blood pressure and diastolic blood pressure measurements were performed on the arm of seated participants after 10 minutes of resting using standard mercury sphygmomanometer. The measurement was repeated after 15 minutes and the average was reported. We calculated eGFR using the Cockcroft and Gault equation, based on age, sex, weight, and serum creatinine.^[25]^

After 12 hours of fasting, venous blood samples were collected for the biochemical analysis. FBS and 1hppg were measured by the glucose oxidase method. HbA1c was measured by high-performance liquid chromatography. Measurement of serum creatinine was performed by Jaffe method. Plasma total cholesterol, triglyceride, high-density lipoprotein cholesterol (HDL), low-density lipoprotein cholesterol (LDL) were determined using direct enzymatic method (Parsazmun, Karaj, Iran).

### 2.3 Outcome measures and definitions

The primary outcome of this study is PP, which is defined as the difference between SBP and DBP. We calculated PP using SBP and DBP, measured at the baseline and during each follow-up.

The diagnosis of DM2 was made based on fasting blood glucose >126 or 2-hour postprandial glucose >200 or randomized blood glucose >200 or HbA1c >6.5.^[26]^ People with hypertension were defined as those who were taking antihypertensive medications. BMI was computed as weight in kilograms divided by height per square meter (kg/m^2^).

### 2.4 Statistical analysis

Statistical analysis was carried out using Stata (version 12; Stata Corp LP, College Station, TX) for Windows. Baseline patients’ characteristics were presented as mean (SD) for continuous variables and number (percentage) for categorical variables. Chi-squared test or one-way ANOVA was used as appropriate to evaluate group differences.

To compare PP, SBP, and DBP among treatment groups, regression models with generalized estimating equation method were used taking into account the correlation between repeated blood pressure measurements of the same subjects. Treatments as indicator variables were main predictors of the model. In the model, the variable “time” was defined as months since the baseline visit. Following, chi-squared test was carried out for comparing treatments’ effect in general. For comparing trends in blood and pulse pressure variation over time in each group, the interaction of treatments and time was compared with and without adjustment for covariates by introducing their product term in regression models with generalized estimating equation method. Age, duration of diabetes, BMI, sex, and smoking were selected as covariates. Post hoc analysis was used as appropriate for pairwise comparison following regression analysis with Sidak correction.

In order to assess change in pulse and blood pressure over time, regression models with generalized estimating equation method were used; in which PP, SBP, and DBP in each studied groups were compared among visits by adjusting for duration of diabetes.

The sample size was calculated assuming 5 mmHg as the least detectable variation of pulse and blood pressure (equivalent of 0.36 standard deviation), type 1 error of 0.05, and 0.5 as the correlation among our repeated measures. Although here by having the least total sample size of 120 as the number of patients in the last visit, this study offers the power of 0.99.

A *P* value <.05 (2-sided) was set as the significance threshold.

## 3 Results

### 3.1 Patients

Seven hundred eighteen diabetic normotensive patients using metformin (189 patients), metformin and glibenclamide (360 patients), metformin and insulin (63 patients), or insulin only (106 patients) were selected from the running cohort study. One hundred seventy two of 718 subjects were selected for the second visit; others were excluded due to either lack of the data or change in the antidiabetic agent. Similarly, the third visit included 138 and the last visit consisted of 120 subjects. The median time gap between our baseline visit and the first, second, and then last follow-up were 12, 23, and 35.5 months, respectively. October 2015 was set as the end of the follow-up.

### 3.2 Baseline characteristics

Table 1 shows baseline characteristics in each studied group. The median age of the study sample was 45.5 years. There was no sex composition difference among studied groups. The baseline PP had no significant variation among study groups. At the baseline, patients on Glb+Met treatment had higher SBP and patients treated with Glb+Met had higher DBP compared with patients using insulin (95% confidence interval [CI] = 0.40–11.36, *P* = .028; 95%CI = 0.59–7.50, *P* = .012, respectively). There was no statistically significant difference among study groups regarding the proportion of smokers.

**Table 1:**
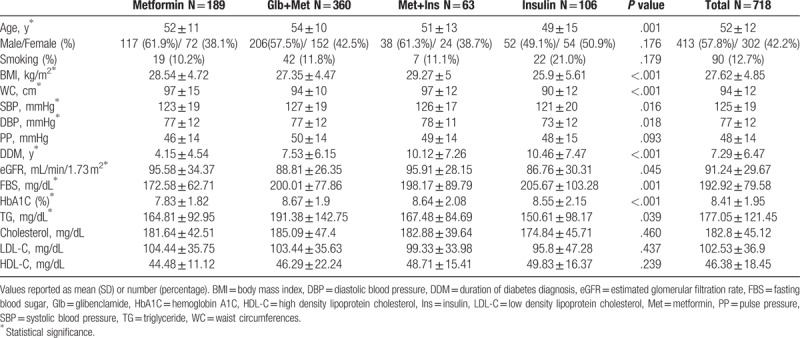
Baseline characteristics of studied groups.

### 3.3 PP, SBP, and DBP change over time in each group

**Figure 1:**
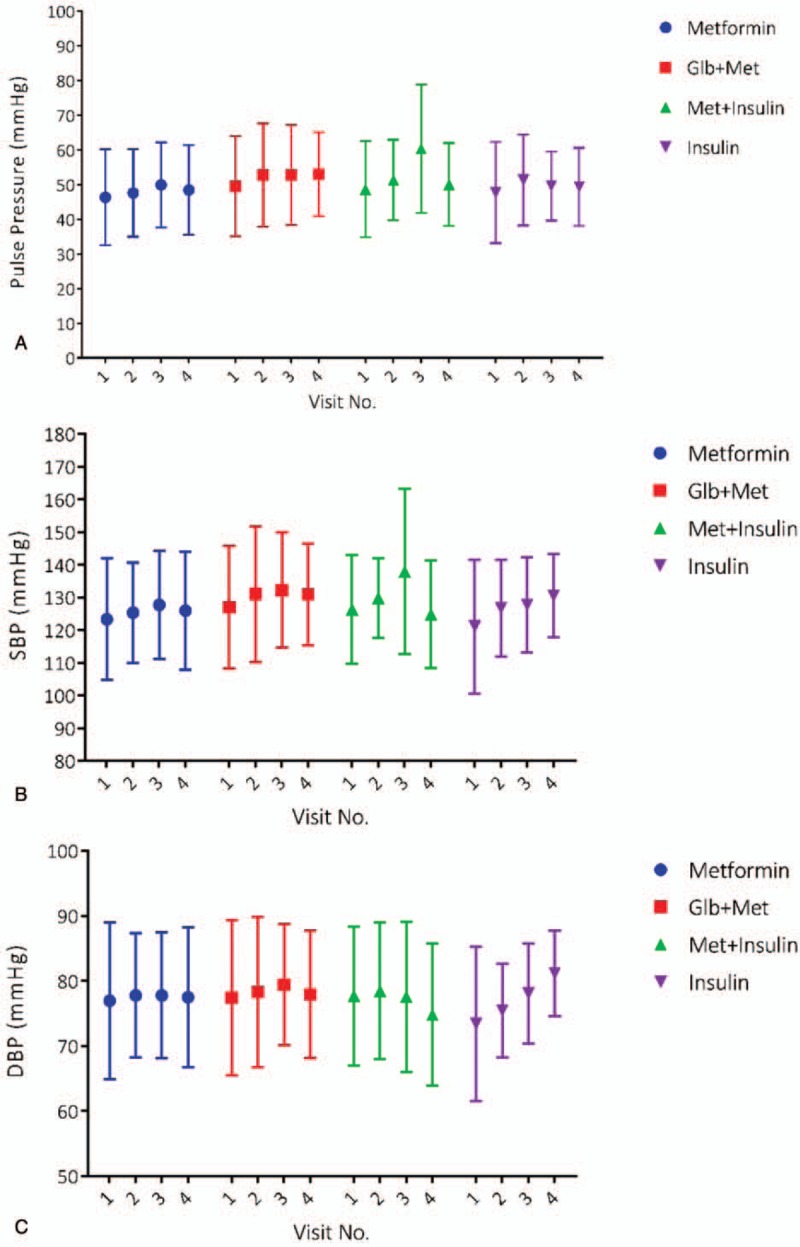
PP, SBP, and DBP of patients in each visit are illustrated in plots (A)–(C), respectively. DBP = diastolic blood pressure, Glb = glibenclamide, Met = metformin, PP = pulse pressure, SBP = systolic blood pressure.

PP, SBP, and DBP of patients over visits are shown in Table 2 and illustrated in Fig. 1A–C. Despite steady blood pressure of other groups, PP (*Χ*^2^ = 13.47; *P* = .003) and SBP (*Χ*^2^ = 8.45; *P* = .037) of patients treated with Met+insulin increased over time. Notably, in this group, PP and SBP alteration were limited to the contrast between first and third visit (PP: 95%CI = 1.38, 19.34; *P* = .014; SBP: 95%CI = −1.32, 22.30; *P* = .111) which did not define a specific change. Notably, including normotensive patients who got hypertensive later had no statistically significant result in this comparison.

**Table 2:**
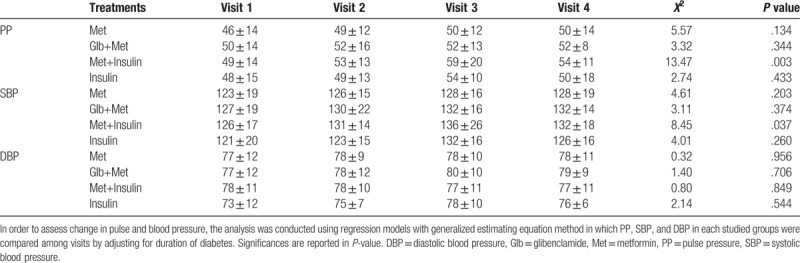
PP, SBP, and DBP among studied groups at each visit.

### 3.4 PP, SBP, and DBP comparison among studied groups

As shown in Table 3, without adjustment PP (*P* < .05), SBP (*P* < .01), and DBP (*P* < .05) all had significant differences among studied groups. After adjustment for covariates variation of PP among groups disappeared (*P* = .090); but, the difference in DBP remained with a trend to statistical significance (*P* = .063) and SBP still varied among studied groups (*P* < .05). Pairwise comparison of PP and SBP among studied groups showed no significant contrast between any 2 groups; but revealed higher DBP in Glb+Met group compared with the insulin group with a trend to statistical significance (95% Cl: −6.26, 0.31; *P* = .098). Table 4 shows the complete pairwise comparison of pulse and blood pressure among studied groups.

**Table 3:**
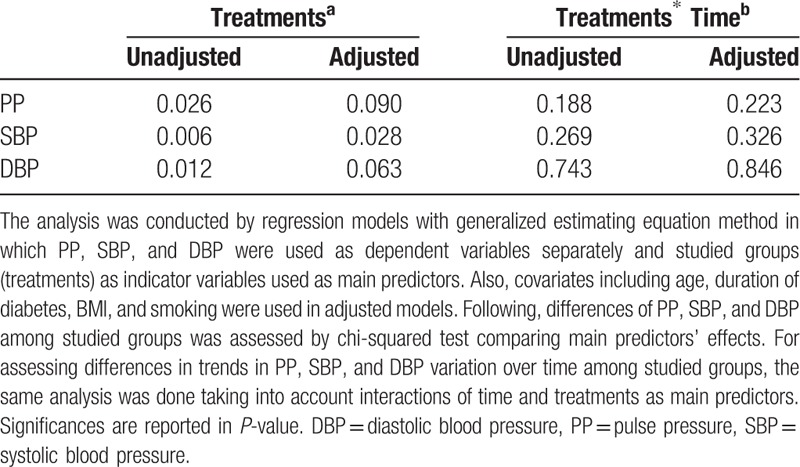
Results of comparison of PP, SBP, and DBP among studied groups.

**Table 4:**
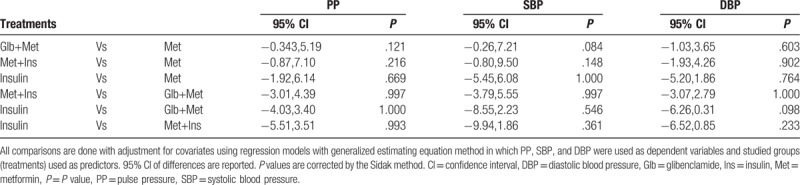
Results of pairwise comparison of studied groups regarding PP, SBP, and DBP.

With and without adjustment, treatments and time had no interaction in predicting PP, SBP, and DBP which showed trends in blood and pulse pressure variation over time among studied groups had no difference. Again including normotensive patients who got hypertensive later had no statistically significant result in these comparisons.

## 4 Discussion

Higher PP in DM2 increases the risk of CVD and its mortality.^[3,6,27]^ DM2 is associated with metabolic syndrome and other cardiovascular risk factors; hence, cardiovascular safety of different glucose-lowering therapies is a critical issue and several studies have already been done comparing cardiovascular outcomes of different antidiabetic treatments.^[28–31]^

There have not been enough studies comparing the outcome of different modalities of glucose-lowering therapy regarding PP. In this study, we have demonstrated pulse and blood pressure of DM2 patients treated with classic glucose-lowering treatments including Met, Glb+Met, Met+insulin, and insulin had no significant change over time. Importantly, we have shown there was no difference in PP, SBP, and DBP among patients treated with these medications.

Many studies have reported metformin reduces cardiovascular events and mortality compared with other antidiabetic agents.^[29,32,33]^ However, the role of blood pressure in this risk reduction has not been elucidated yet. Although some authors have reported a decrease in SBP or DBP by using metformin,^[34,35]^ there are studies reporting a nonsignificant effect of this medication on patients blood pressure.^[36,37]^ We did not find any significant change of PP, SBP, and DBP in patients treated with metformin over follow-up. There have not been enough studies on the effect of metformin on PP; however, Wulffelé et al^[37]^ demonstrated a decrease in nocturnal PP of diabetic patients including both normotensive and hypertensive cases after 16 weeks treatment with metformin.

It seemed PP and SBP of patients treated with Met+insulin increased over time, but this variation was only between first and third visit which could not reliably define a trend. Formerly, Mourão-Júnior et al^[38]^ demonstrated the addition of metformin to diabetic patients controlled with insulin had no effect on blood pressure. Studies should be done for clarifying the effect of adding insulin to metformin or vice versa on blood pressure and most importantly pulse pressure.

Among mechanisms in which sulfonylurea could lead to the higher CVD morbidity, Williams has shown glibenclamide increases nocturnal SBP in diabetic patients. We did not find any study assessing mainly the effect of sulfonylurea on PP in DM2; although St John Sutton et al^[44]^ provided data showing a statistically significant decrease in diabetic patients PP after 52 weeks treatment with Glyburide. Also, we observed no change in SBP, DBP, and PP of diabetic patients treated with Glb+Met. Similarly, Herman et al^[47]^ had found no blood pressure difference among patients treated with different combinations of metformin and Glyburide. Again, there has been a lack of studies about the effect of Glb+Met combination treatment on PP.

There are studies reporting increased cardiovascular events and mortality by insulin treatment in DM2 patients especially by applying intensive blood glucose control.^[49–51]^ On another hand, some studies have reported an insignificant association between insulin use and cardiovascular morbidity and mortality.^[52,53]^ Here we focused on the role of pulse and blood pressure and found no significant change in SBP and DBP of diabetic patients treated with insulin; however, DBP in patients treated with insulin in this study was lower than patients on Glb+Met with a trend to statistical significance. There are studies showing blood pressure elevation secondary to insulin initiation in diabetic patients^[21,19]^;however, our finding is consistent with Flores et al^[20]^ which reported no rise in blood pressure secondary to insulin therapy. Furthermore, Rowe et al^[54]^ had already demonstrated insulin infusion could lead to increase in PP; although, we observed no difference in PP of patients treated with insulin over the follow-up.

As noted before, there have not been enough studies comparing these classic glucose-lowering treatments according to their effect on PP. Skov et al^[24]^ already demonstrated that effect of different combinations of insulin, metformin, or Rosiglitazone on PP did not vary. We also have shown there is no difference in trends of PP, SBP, and DBP variation over time among studied groups which has not been reported before.

In our study, after adjustment for covariates and pairwise comparisons, no significant contrast between any 2 groups was observed. However, in crude analysis SBP, DBP, and PP were different among treatment groups. The effect of adjustment showed covariates confounding effects and could be explained by following facts. First, with prolonged duration of DM2, vessels would be exposed to hyperglycemic media longer which facilitates arterial stiffening. Also, duration of diabetes itself has shown to be associated with pulse pressure.^[55]^ Second, hypoglycemic medications such as metformin are selected based on patients’ characteristics such as BMI^[56]^ which is formerly reported to have a positive association with pulse pressure.^[57]^ Together, different BMI, DM2 duration, and age of patients among this study groups could lead to a significant crude result.

This study is among few studies comparing PP of diabetic patients treated with different antidiabetic regimens. Our study is highlighted by having a large and targeted sample size and using generalized estimating equation analysis. Notably, we excluded hypertensive patients as well as patients treated with lipid-lowering therapies other than statins due to the interference their various treatments could cause. Including normotensive people who got hypertensive later along follow-up had no statistically significant outcome, showing this exclusion had not biased the final result. However, we had some limitations. First, studied groups were not randomized; although, we adjusted results for some known covariates to compensate for the lack of randomization in this prospective cohort study. Furthermore, randomized clinical trial (RCT) design limits the follow-up time and restricts trends study of pulse and blood pressure, an analysis requiring long follow-up period. Second, we had no control group to compare treatment groups with; consequently, we were not able to demonstrate each drug net effect on patients’ blood pressure. Although we evaluated and compared the effect of 4 antidiabetic medication regimen on pulse pressure over time, further studies needed to investigate other cardiovascular safety issues.

## 5 Conclusion

There was no statistically significant difference in PP, SBP, and DBP among patients treated with classic antidiabetic regimens including metformin, Glb+Met, Met+insulin, and insulin alone. Trends in PP, SBP, and DBP variation over time were not different among studied glucose lowering modalities. These hypoglycemic regimens also did not affect PP, DBP, and SBP over time. However, further studies are needed to improve our understanding of these findings.

## Uncited references

^[39–43]^, ^[45]^, ^[46]^, ^[48]^.
